# Multi-Layer Electrospun Membrane Mimicking Tendon Sheath for Prevention of Tendon Adhesions

**DOI:** 10.3390/ijms16046932

**Published:** 2015-03-26

**Authors:** Shichao Jiang, Hede Yan, Dapeng Fan, Jialin Song, Cunyi Fan

**Affiliations:** 1 Department of Orthopaedics, Shanghai Jiaotong University Affiliated Sixth People’s Hospital, 600 Yishan Road, Shanghai 200233, China; E-Mails: mailjsc@163.com (S.J.); niaodoctor@163.com (D.F.); songjialinsjtu@126.com (J.S.); 2 Department of Orthopaedics, the Second Affiliated Hospital of Wenzhou Medical University, 109 West Xueyuan Road, Wenzhou 325027, China; E-Mail: yanhede@hotmail.com

**Keywords:** tendon adhesions, multi-layer, hyaluronic acid, celecoxib, electrospun, PELA

## Abstract

Defect of the tendon sheath after tendon injury is a main reason for tendon adhesions, but it is a daunting challenge for the biomimetic substitute of the tendon sheath after injury due to its multi-layer membrane-like structure and complex biologic functions. In this study, a multi-layer membrane with celecoxib-loaded poly(l-lactic acid)-polyethylene glycol (PELA) electrospun fibrous membrane as the outer layer, hyaluronic acid (HA) gel as middle layer, and PELA electrospun fibrous membrane as the inner layer was designed. The anti-adhesion efficacy of this multi-layer membrane was compared with a single-layer use in rabbit flexor digitorum profundus tendon model. The surface morphology showed that both PELA fibers and celecoxib-loaded PELA fibers in multi-layer membrane were uniform in size, randomly arrayed, very porous, and smooth without beads. Multi-layer membrane group had fewer peritendinous adhesions and better gliding than the PELA membrane group and control group in gross and histological observation. The similar mechanical characteristic and collagen expression of tendon repair site in the three groups indicated that the multi-layer membrane did not impair tendon healing. Taken together, our results demonstrated that such a biomimetic multi-layer sheath could be used as a potential strategy in clinics for promoting tendon gliding and preventing adhesion without poor tendon healing.

## 1. Introduction

The tendon sheath, which surrounds the tendon in some areas of the hand and foot, is an important structure for tendon gliding and nutrition. It consists of an outer fibrous sheath and an inner synovial sheath, which are two thin and serous sheets, the parietal and visceral sheets. These sheets form a closed duct, including peritendinous fluid (mainly hyaluronic acid, HA) for lubrication [1,2]. Additionally, the tendon sheath itself is a good biological barrier that prevents invasion of peripheral fibrous tissue and promotes tendon healing [3]. When tendon injury happened, the tendon sheath was always impaired or defected. Thus, invasion of exogenous fibroblasts allowed the surrounding tissue to attach to the repair site and resulted in adhesion formation. To avoid the daunting complication, a tendon sheath-like structure is essential for tendons to perform normally.

Nowadays, a great deal of materials, such as HA and HA-membrane [4,5,6], solvent dehydrated bovine pericard [7], and polyvinyl alcohol-hydrogel [8], have been applied for tendon sheath repair or reducing tendon adhesion. However, none of them were satisfied with a tendon sheath-like structure, a multi-layer sheath structure. In addition, an ideal substitute for the tendon sheath should not only mimic the structure of tendon sheath but also inhibit invasion of exogenous fibroblasts and perform the biological function of secreting synovial fluid, which can decrease adhesion and facilitate tendon repair.

Electrospinning, as an effective technique, had numerous applications in biomedicine. Many electrospinning techniques like coaxial electrospinning [9], emulsion electrospinning [10], and mixing electrospinning [11] have been applied to fabricate polymer nanofibers for tissue repair or anti-adhesion. However, achieving emulsion electrospinning and coaxial electrospinning was technically demanding due to the spinneret complexity and potential instability of coaxial flow upon feeding. Thus, sequential electrospinning may be a simple and reliable technique.

In previous studies, the electrospun poly(l-lactic acid)-polyethylene glycol (PELA) diblock copolymer fibrous membrane has been reported to prevent adhesions due to its favorable hydrophilic properties and degradation patterns [12], but its anti-adhesion ability was limited [11]. Celecoxib, as a kind of selective non-steroidal anti-inflammatory drugs (NSAIDs), can suppress fibroblast proliferation and collagen expression by inhibiting ERK1/2 and SMAD2/3 phosphorylation [13]. Additionally, in our previous study, a celecoxib-loaded PELA diblock copolymer fibrous membrane has been fabricated by electrospinning [14]. The results indicated that a burst release was followed by sustained release from fibrous membranes with high initial celecoxib content. Fewer cells adhered to and proliferated on the celecoxib-loaded PELA fibrous membrane *in vitro* and the celecoxib-loaded PELA fibrous membrane significantly prevented tissue adhesion with impairing tendon healing to some extent *in vivo*.

In this study, a multi-layer electrospun membrane-like tendon sheath structure was designed, the outer layer was celecoxib-loaded PELA electrospun fibrous membrane, the inner layer was PELA electrospun fibrous membrane, and HA gel was placed between two layers. The purpose of this study was to evaluate whether this multi-layer electrospun membrane can prevent adhesion by the outer layer and promote tendon gliding and healing by HA and the inner layer.

## 2. Results and Discussion

### 2.1. Characterization of Multi-Layer Electrospun Fibrous Membrane

**Figure 1:**
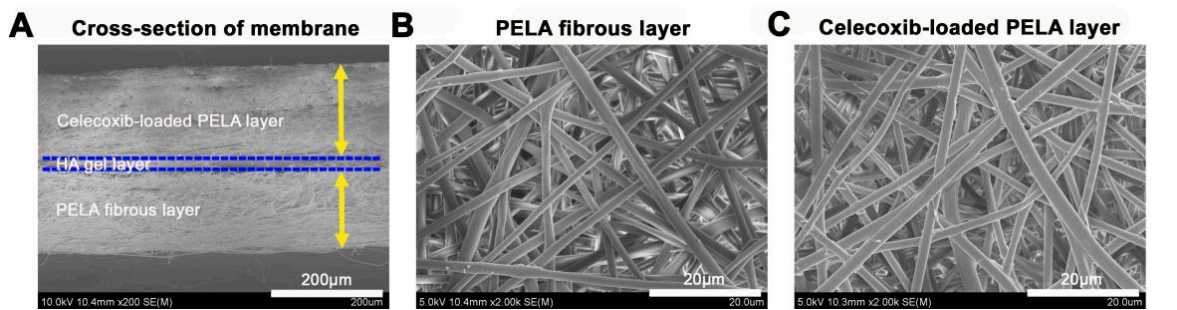
Scanning electron microscopy (SEM) observation for cross-sectional features of the multi-layer membrane (**A**) and surface morphological features of the poly(l-lactic acid)-polyethylene glycol (PELA) electrospun fibers (**B**) and celecoxib-loaded PELA electrospun fibers (**C**).

Both surface morphology of the multi-layer electrospun fibrous membrane were shown. From cross-sectional morphology of Figure 1A, we could learn that thicknesses of the multi-layer membrane were 130 µm for the PELA layer and 170 µm for the outer celecoxib-loaded PELA layer. As shown in Figure 1B,C, both PELA and celecoxib-loaded PELA fibers were uniform in size, randomly arrayed, very porous and smooth, without beads in the fibrous structure.

### 2.2. In Vivo Animal Study

**Figure 2:**
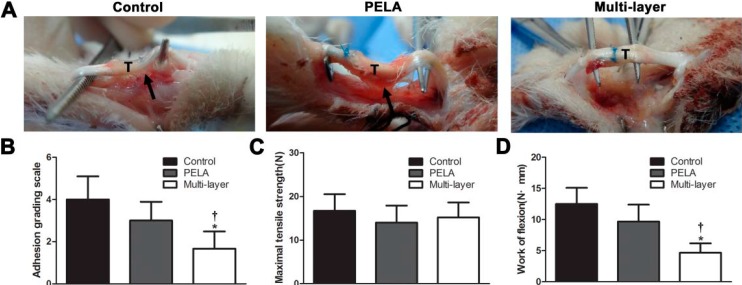
(**A**) Gross evaluation of the rabbit flexor digitorum profundus (FDP) tendon model in the untreated control group, PELA membrane group, and multi-layer membrane group. Tendon (T) is indicated in the figures and adhesion tissue is pointed to using black arrows. Tendon repair and peritendinous adhesions are evaluated by determining macroscopic evaluation of tendon adhesions (**B**), maximum tensile strength (**C**), and work of flexion (**D**). * *p* < 0.05 compared with control group; **^†^** *p* < 0.05 compared with PELA membrane group. Data are expressed as mean ± SEM for six tendons/group.

After three weeks, the animals were killed, the toes were dissected through an incision on the volar aspect. The wounds appeared macroscopically free of infection or ulcer. The adhesions observed between the tendon and surrounding tissues were evaluated by the adhesion grading system. In the control group there were severe peritendinous adhesions around the repair site that required sharp dissection to separate. Unloaded PELA membrane resulted in small bundles of fibrous tissues bridging the tendon and the surrounding tissue, but in multi-layer membrane group, few adhesions were observed between the repaired tendon and the surroundings and a spatula could easily go through under the tendon (Figure 2A). There were significantly fewer adhesions in the multi-layer membrane group than in the control group and PELA membrane group (*p* < 0.05) (Figure 2B).

Work of flexion and maximum tensile strength for evaluating peritendinous adhesions and tendon healing were summarized in Figure 2. The maximum tensile strength of the control group was higher than that of the PELA fibrous membrane group and multi-layer membrane group, but there was no significant difference in maximum tensile strength among three groups (Figure 2C). Work of flexion was significantly lower in the multi-layer membrane group than the control group and PELA fibrous membrane group (*p* < 0.05) (Figure 2D).

**Figure 3:**
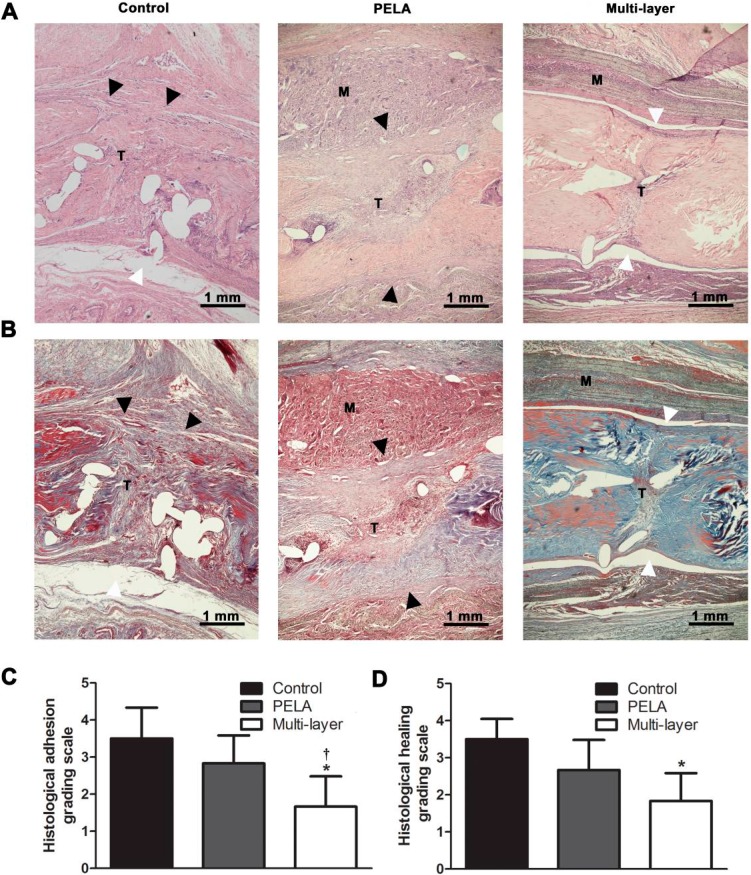
HE (**A**) and Masson (**B**) staining of untreated repair site, repair sites wrapped with unloaded PELA fibrous membrane and wrapped with a multi-layer fibrous membrane. White arrowheads indicate the interface without peritendinous adhesions, while black arrowheads indicate peritendinous adhesions between the membrane (M) and tendon (T) (Scale bar = 1 mm). Peritendinous adhesions and tendon repair are evaluated by determining histological adhesion grade (**C**) and histological healing grade (**D**). * *p* < 0.05 compared with control group; **^†^** *p* < 0.05 compared with PELA membrane group. Data are expressed as mean ± SEM for six tendons/groups.

The detailed histological findings regarding peritendinous adhesion formation were presented in Figure 3. In the control group, there were dense adhesions observed between the tendon and the surrounding vascular granulation tissue. Loose bundles of fibrous tissue bridging the repaired tendon and the undegraded membrane were shown in the repaired tendon of the unloaded PELA fibrous membrane group. In comparison, few peritendinous adhesions were detected in most specimens of the multi-layer membrane group (Figure 3A,B). The decrease in the amount of adhesion tissue in the multi-layer membrane group was significantly higher compared with the control group and PELA fibrous membrane group (*p* < 0.05) (Figure 3C). In addition, tendon healing could be indicated by histological findings. Invasion of the fibrous adhesion tissue into the repaired tendons could be observed with poor collagen maturation in the control group. The surfaces of the repaired tendons wrapped with PELA membrane were also very rough, which indicated partial tendon healing. At the repaired sites wrapped with the multi-layer membrane, the surfaces of the tendons were smooth, and well-organized collagen in these tendons was shown, indicating better tendon healing (Figure 3A,B,D).

### 2.3. Collagen Expression in Repair Site

**Figure 4:**
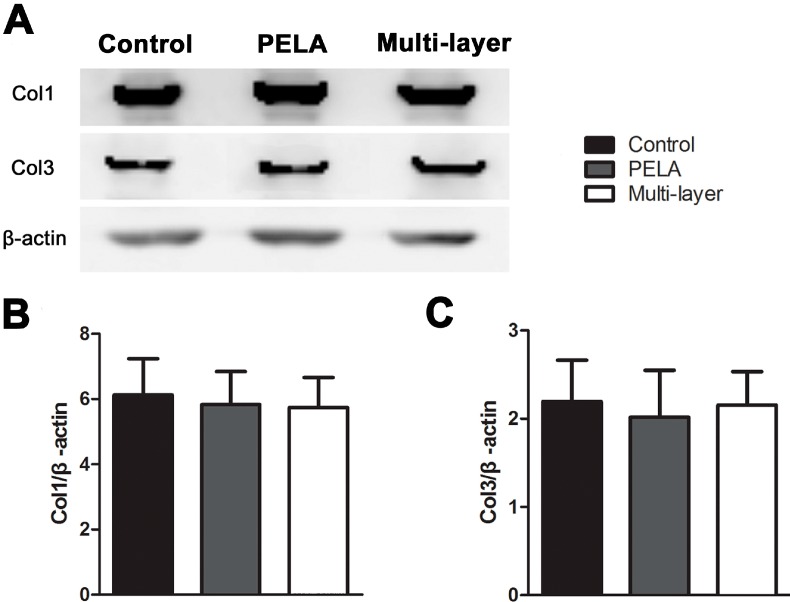
Western blotting assay for collagen I and collagen III expression in repair sites with the untreated group, PELA fibrous membrane group, and multi-layer fibrous membrane group for three weeks (**A**). Densitometry of collagen I (**B**) and collagen III (**C**) from Western blot experiment. Data are expressed as mean ± SEM for six tendons/group.

Collagen expression of tendon repair site analyzed by Western blotting assays also indicated the healing effect of the repair site treated with different membranes. The protein levels of collagen I and collagen III in the repair sites after three weeks were examined, with β-actin as protein loading control. As shown in Figure 4, collagen I and collagen III expression were low in the experimental groups compared with the control group, but there was no significant difference among three groups. This meant that multi-layer membrane did not affect the collagen synthesis of tendon healing.

### 2.4. Discussions

In previous studies, many materials and methods, such as hydrogel [15], HA membrane [16], PELA membrane [11], and human amniotic membrane [17], were used in an attempt to reduce tendon adhesion. However, these materials and methods were used individually and could not mimic the structure and function of a tendon sheath, which not only prevented adhesion but also provided tendon the nutrition, so the ability of anti-adhesion was limited. To avoid the limited ability, some researchers [10] discovered a electrospun *bi*-layer membrane consisting of hyaluronic acid-loaded poly(ε-caprolactone) (HA/PCL) fibrous membrane as the inner layer and a PCL fibrous membrane as the outer layer was fabricated by a combination of sequential and microgel electrospinning technologies. However, emulsion electrospinning and coaxial electrospinning had some difficulties in the techniques due to the spinneret complexity and potential instability of coaxial flow upon feeding. Therefore, this method limited its application. In a previous study, we discovered a celecoxib-loaded PELA electrospun fibrous membrane and revealed that it prevented tissue adhesion significantly by inhibiting ERK1/2 and SMAD2/3 phosphorylation, but this strategy partly impaired the intrinsic healing of tendon repair [14]. It has been proven that NSAIDs has potential side effects, such as gastrointestinal side effects, and renal and arrhythmia risks. However, celecoxib had a greater safety and lower risk than any other NSAIDs. Furthermore, celecoxib was mixed into PELA with a very low dose and used locally, this method minimized the side effect of celecoxib and provided more safety and reliability for further clinical use.

**Figure 5:**
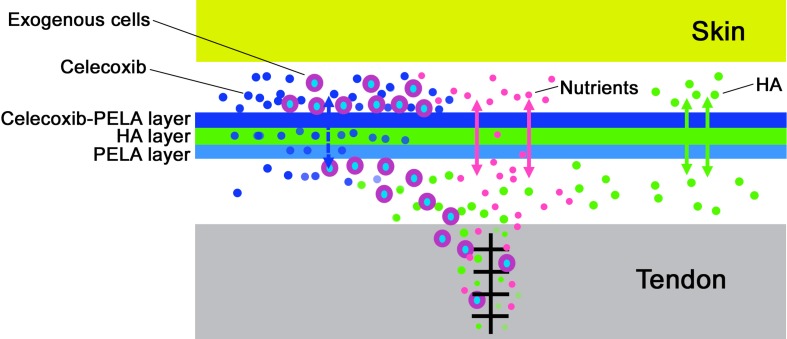
Schematic illustration of multi-layer membrane as physical barrier for preventing tendon adhesions after tendon injury.

In this study, in order to discover a strategy, which not only prevents adhesions, but also protects tendon healing, we fabricated a multi-layer structure with celecoxib-loaded PELA electrospun fibrous membrane as the outer layer, HA gel as the middle layer and PELA electrospun fibrous membrane as the inner layer to replace the native tendon sheath. As shown by SEM (Figure 1), such a multi-layer membrane has a structure mimicking the structure of a native tendon sheath, which consists of an outer fibrous sheath, peritendinous fluid layer, and inner synovial sheath. There are many advantages for this discovery. First, sequential electrospinning is a very simple electrospun technology and we can simply fabricate the multi-layer membrane without considering the spinneret complexity and potential instability of coaxial flow on microgel electrospinning. Second, the celecoxib-loaded PELA electrospun fibrous membrane, which is located as the outer layer, can not only prevent cell invasion like the native fibrous sheath, but also significantly inhibit exogenous fibroblasts proliferation and collagen synthesis, which the native fibrous sheath does not [14]. The efficiency of the anti-adhesion effect has been supported by gross view and histological assessments. As the middle layer, on the one hand, HA gel has a buffer action for preventing celecoxib, which is released from outer layer, impairing intrinsic healing and, on the other hand, acts as a kind of lubricant and nutrient. The PELA membrane, as the inner layer, mainly acts as a physical barrier and maintains tendon gliding with its high hydrophilicity and smoothness. Furthermore, intrinsic tendon healing can be promoted by diffusion of nutrients from outside the sheath to the repair site through the very small pores of the PELA membranes (Figure 5). According to the *in vivo* results of our study, macroscopic and histological evaluation and work of flexion indicated good gliding, achieved with the HA gel layer and PELA fibrous layer. In addition, collagen I and collagen III synthesis was a main process in the remodeling phase of tendon healing after repair [18], thus, the synthesis and remodeling of collagen I and collagen III was critical to tendon healing. The statistical indifference of maximum tensile strength and collagen expression of the tendon repair site in the three groups indicated that the multi-layer membrane had the ability of protecting intrinsic healing.

At present, many studies focused on engineered tendon sheath [19,20,21], although tissue engineering of a tendon sheath was thought to be an ideal substitute for the native tendon sheath, seeding cells onto these scaffolds to produce HA-secreting sheaths had limited therapeutic potential because of the limited supply of seed cells, immunological rejection with allograft cells, and donor site defects in healthy compartments had to be considered as potential disadvantages for clinical applications.

Our novel multi-layer membrane was very suitable for the clinical reality of anti-adhesion because no synovial cells for *in vitro* culture and expansion were required. Restoration of sheath integrity was considered to provide a smooth gliding surface for tendons [22], decrease peritendinous adhesions, and preserve nutrition for the tendons, but the tendon sheath was difficult to repair. Consequently, our “dead” multi-layer membrane provided a therapeutic opportunity, which mimicked the biological function and structure of the native sheath, especially under the condition of defect of the tendon sheath. In addition, our sheath membrane could reinforce the ability of anti-adhesion by celecoxib-loaded PELA fibrous membrane, compared with the native sheath. Although three weeks was the critical period when tendon healing was achieved and rehabilitation exercises were started in the clinic, and it was sufficient for a preliminary investigation of such a membrane, the later effects on tendon healing and anti-adhesion after the complete degradation of the multi-layer membranes have not been proven. Thus, it will be focused on in our future studies.

## 3. Experimental Section

### 3.1. Materials

Celecoxib was purchased from TSZ Co. (Framingham, MA, USA). HA polymer sourced from human umbilical cords was purchased from Sigma-Aldrich (St. Louis, MO, USA). Other reagent grade chemicals and solvents except otherwise indicated were purchased from GuoYao Regents Company (Shanghai, China).

### 3.2. Electrospinning of Nanofibrous Membranes

Electrospinning was carried out according to our previous report [14]. First, 1 g PELA (*M*_W_ = 40 kDa, *E*/*L* = 10/90, *M*_W_/*M*n = 1.56) and 0.06 g celecoxib were completely dissolved in a solvent containing 2.1 g dichloromethane (DCM) and 1.1 g dimethylformamide (DMF) for preparing the celecoxib-loaded PELA electrospun fibrous membranes. Then, we used a 0.7 mm diameter needle, fitted to a 2.0 mL glass syringe and a syringe pump, for preparing the celecoxib-loaded PELA membrane. The mixture was electrospun with the following parameters: applied voltage was 15 kV; flow rate was 3.0 mL/h; and spin length was 15 cm. The electrostatic force drew the polymeric solution from the needle tip to reach the collector that was placed away from the needle tip. The outer layer of the multi-layer electrospun fibrous membrane was first fabricated by co-electrospinning. Then, 1 wt. % HA hydrosols, which were 10 mg HA dissolved in 990 mg distilled water, were covered on the celecoxib-loaded PELA membrane. Finally, 1 g PELA was dissolved in a solvent containing 2.1 g DCM and 1.1 g DMF and a PELA membrane was collected on an HA gel membrane by sequential electrospinning. The fabricated fibrous membranes were dried overnight in a vacuum oven before further use. The morphology of the membranes was observed by scanning electron microscopy (SEM, FEI Quanta 200, Eindhoven, The Netherlands).

### 3.3. Preliminary Animal Study

Seventy-two adult New Zealand White rabbits (weight range, 2.0–3.0 kg) were used as model animals (protocol approved by Shanghai Jiao Tong University School of Medicine and the National Institutes of Health). After being anesthetized with an intravenous injection of pentobarbital sodium (50 mg/kg), and sterile skin preparation, an elastic tourniquet was applied. The lateral aspect between the metacarpo-phalangeal and proximal inter-phalangeal joints of the middle digit was exposed by a 1.5-cm linear skin incision and separation of the subcutaneous tissue. The flexor tendon sheath was incised, and the flexor digitorum superficialis (FDS) tendon was removed. A full-thickness transverse tenotomy was made with a number 15 blade on the flexor digitorum profundus (FDP) tendon, then the ruptured tendon was repaired using a modified Kessler tendon repair with 6-0 prolene suture (Ethicon Ltd., Edinburgh, UK). The tendon sheath was not repaired. The animals were randomly divided into three groups (control and two experimental groups) with twenty-four animals per group. A 1 cm × 1 cm piece of PELA electrospun membrane was wrapped around the repair site of the FDP tendon of each animal in one experimental group and a multi-layer membrane of the same size was wrapped around the repair site of the FDP tendon of each animal in the other experimental group, while the control group received no treatment around the repair site before closure. After the final skin closure, the extremity was immobilized in a self-made splint.

### 3.4. Macroscopic Evaluation

The animals were killed after 3 weeks. First, the repaired site was examined for any sign of inflammation or ulcer. Severity and extent of peritendinous adhesion were scored, respectively. For the quantitative macroscopic evaluation of the peritendinous adhesions, the adhesion scoring system was used at a particular area into grade 1–5, based on the surgical findings (grade 1, no adhesions; grade 2, filmy adhesions easily separable by blunt dissection; grade 3, less than or equal to 50% of the adhesion areas must be separated by sharp dissection; grade 4, 51%–97.5% of the adhesion areas must be separated by sharp dissection; and grade 5, more than 97.5% of the adhesion areas must be separated by sharp dissection) [23]. The adhesion rate was used to quantify the extent of peritendinous adhesions [24]. Adhesions were evaluated by two independent investigators blinded to treatment.

### 3.5. Histological Evaluation

The middle toes were collected and fixed in 4% paraformaldehyde for 2 days and decalcified for 1 month with 10% EDTA at room temperature. After being dehydrated in an ascending alcohol series and embedded in paraffin, 4-μm sagittal sections were cut and stained with hematoxylin and eosin (HE) and Masson’s trichrome. Histologic assessments of adhesions and tendon healing were performed [25]. Adhesions were quantified into four grades as follows: no adhesions; <33% of the tendon surface; 33%–66% of the tendon surface; >66% of the tendon surface [26]. Tendon healing was quantified into four grades according to Tang *et al.* as follows: grade 4, poor (separation of the sutured parts or mass overgrowth of granulation); grade 3, fair (irregular intratendinous collagen bundles, and partly interrupted by adhesions); grade 2, good (intratendinous collagen bundles exhibited good repair, but the epitenon was interrupted by adhesions); or grade 1, excellent (continuity of tendon was re-established and the epitenon was smooth) [27]. These histological sections were evaluated microscopically (LEICA DM 4000 B; Leica Microsystems, Bannockburn, IL, USA) by two independent observers blinded to the treatment.

### 3.6. Biomechanical Evaluation

The FDP tendon of the middle toe was exposed at the ankle for the biomechanical test. The work of flexion and maximal tensile strength were measured using a rheometer (Instron 5548, Instron, MA, USA). The proximal end of the FDP tendon was fixed to a force gauge, and the proximal phalanx of the toe was attached to a self-made device with K-wires through the distal interdigital joint. To evaluate the work of flexion, the actuator pulled the tendon at 10 mm/min until the range of proximal interdigital joint reached 40°. The force and the excursion of the clamp were measured directly, and the work of flexion was calculated by curve integration. Then, the proximal and distal ends of the tendon were grasped with the compressive clamp attached to the rheometer. The device pulled the tendon at 10 mm/min, until the tendon ruptured. The breaking strength at the repair site represented the maximal tensile strength. In this case, the maximal tensile force was recorded.

### 3.7. Western Blot Analysis

Western blot was used to quantify protein levels of collagen I and collagen III in the repair site. Total protein was isolated from tissues of the repair site by homogenization in a buffer containing 200 µL of ice-cold RIPA (Bio-Rad, Hercules, CA, USA) and 1 µL of 200 mM PMSF (Kang Chen Corp., Shanghai, China) on ice for 60 min. Then, the lysates were centrifuged at 12,000 rpm for 10 min. The supernatants were collected and the total protein content was measured by the BCA protein assay kit (Thermo, Rockford, IL, USA). Equal amounts of sample proteins were electrophoresed through an 8% SDS-PAGE gel, and then transferred to a polyvinylidene difluoride membrane (Millipore, Bedford, MA, USA). After blocking with 5% non-fat milk in TBST buffer (50 mm Tris-HCl, 100 mm NaCl, and 0.1% Tween-20, pH 7.4) at room temperature for 60 min, the membranes were incubated with antibodies against collagen I (Biorbyt, Cambridge, UK), collagen III (Abbiotec, San Diego, CA, USA), and β-actin (Abcam, Cambridge, MA, USA) overnight at 4 °C. After washing with TBST buffer, the membranes were incubated with corresponding horseradish peroxidase-conjugated IgG (Cell Signaling, Beverly, MA, USA) for 1 h. Then, the membranes was washed with TBST buffer three times and detected with an enhanced chemiluminescence detection kit (Thermo Pierce) and an imaging system (Image Quant LAS 4000 mini, GE, Piscataway, NJ, USA). The density of bands was quantified with Image Pro-plus 6.0 (Media Cybernetics, Bethesda, MD, USA).

### 3.8. Statistical Analysis

Data were expressed as mean ± SEM. Statistical software SPSS 13.0 was performed to analyze the data using one-way analysis of variance; values of *p* < 0.05 were considered to be statistically significant.

## 4. Conclusions

In this study, a multi-layer membrane with celecoxib-loaded PELA electrospun fibrous membrane as the outer layer, HA gel as the middle layer, and PELA electrospun fibrous membrane as the inner layer was fabricated by sequential electrospinning. *In vivo* results showed that the outer celecoxib-loaded PELA layer could reinforce the anti-adhesive function of the outer fibrotic layer, while the middle HA gel and inner PELA layer can mimic the biological function of HA secretion to promote tendon healing and gliding. Taken together, these results demonstrated that the multi-layer membrane prevented tendon adhesion formation effectively without impairing tendon healing, and may possibly be used as a prophylactic strategy in the management of patients with tendon adhesions.
